# F71. THE STRUCTURE OF NEUROCOGNITION ACROSS CHILDHOOD AND ADULTHOOD IN YOUNG PEOPLE WITH PSYCHOSIS.

**DOI:** 10.1093/schbul/sby017.602

**Published:** 2018-04-01

**Authors:** Josephine Mollon, Anthony David, Stanley Zammit, Glyn Lewis, Avi Reichenberg

**Affiliations:** 1 Yale University; 2 Institute of Psychiatry, King’s College London; 3 Cardiff University & University of Bristol; 4 University College London; 5 Icahn School of Medicine at Mount Sinai

## Abstract

**Background:**

There is substantial evidence for connection abnormalities in the brains of schizophrenia patients. However, little is known about the structure of cognitive functioning across the psychosis spectrum.

**Methods:**

We used data from the Avon Longitudinal Study of Parents and Children (ALSPAC). Data from all individuals who underwent cognitive testing at age 8 and psychiatric assessment at 18 years were used to examine network structure of cognition in childhood (age 8). A subsample of individuals who underwent further cognitive testing at age 20 was used to examine change in cognitive network structure between childhood (age 8) and adulthood (age 20). Networks comprised nodes (cognitive tests) joined together by edges (partial correlations). Organization of subnetworks by cognitive domains (verbal, perceptual, working memory and processing speed) and measures indicating 1) important cognitive tests or hubs, 2) network integration and 3) network density, were examined. Participants with non-affective psychotic disorder, affective psychotic disorder, psychotic experiences and depression were compared to controls.

**Results:**

In childhood, affective and non-affective psychosis groups showed disruption to cognitive subnetworks and hubs, as well as greater network connectivity (β=0.44, p<.001, β=0.16, p<.001), dysconnectivity (β=−0.47, p<.001, β=−0.19, p=.002), integration (β=−12.7, p<.001, β=−10.2, p<.001) and density (β=0.49, p<.001, β=0.17, p<.001). The psychotic experiences group showed intact subnetworks and hubs, but increased network integration (β=−5.5, p<.001) and density (β=0.02, p=.04). The depression group also showed intact subnetworks and hubs, but increased integration (β=−5.9, p<.001). Between childhood and adulthood increasing density was seen in the psychotic experiences group (β=0.09, p=.04), and the depression group showed increasing integration (β=−3.15, p=.04). Controls showed increasing reliance on the working memory hub between childhood and adulthood, while all other groups remained reliant on attention and visuospatial abilities.

**Discussion:**

Overall, individuals with psychotic disorder showed substantial qualitative and quantitative differences in cognitive network structure. Individuals with psychotic experiences and depression showed more subtle deviations. Abnormalities in cognitive network structure were seen even in the absence of cognitive impairment, suggesting the importance of looking beyond deficits to how performance is achieved.

